# Nicotinic Acid Riboside Regulates Nrf-2/P62-Related Oxidative Stress and Autophagy to Attenuate Doxorubicin-Induced Cardiomyocyte Injury

**DOI:** 10.1155/2022/6293329

**Published:** 2022-02-22

**Authors:** Linfeng Zou, Bing Liang, YuanZhen Gao, Ting Ye, MengJiao Li, Yukun Zhang, Qi Lu, Xiaokun Hu, Huanting Li, Yang Yuan, Dongming Xing

**Affiliations:** ^1^School of Basic Medicine and the Affiliated Hospital of Qingdao University, Qingdao University, Qingdao 266071, China; ^2^Qingdao Cancer Institute, Qingdao 266071, China; ^3^School of Life Sciences, Tsinghua University, Beijing 100084, China

## Abstract

Doxorubicin (Dox) is an effective chemotherapeutic drug for the treatment of various cancers. Due to its potential fatal cardiotoxic side effects, the clinical application is often limited. Dexrazoxane (Dex) is the only drug approved by the Food and Drug Administration (FDA) for the prevention of Dox-induced cardiotoxicity but has side effects. Thus, more protective strategies should be explored. If NAD^+^ plays a role in maintaining heart function, its precursor prospectively alleviates Dox-induced cellular injury. Here, we studied the protective effects of nicotinic acid riboside (NAR) on Dox-induced cardiotoxicity *in vivo* and *in vitro*. We found that NAR significantly improved the cardiac function of Dox-treated mice by restoring ejection fraction (EF), fractional shortening (FS), and serum level of cardiac troponin (cTnI). NAR not only reduced malondialdehyde (MDA), lactate dehydrogenase (LDH), and reactive oxygen species (ROS) levels in Dox-treated cardiomyocytes but also further promoted the activities of cardiac superoxide dismutase (SOD) and glutathione (GSH). Following exposure to 5 *μ*M Dox, cotreatment with NAR exhibited increased cell viability with a decrease in the apoptosis cell population. Moreover, the levels of apoptosis-related proteins, as well as proteins involved in oxidative stress and autophagy, were altered after NAR treatment. Collectively, these findings underline the protective potential of NAR against Dox-induced cardiomyocyte injury by regulating Nrf-2/P62-related oxidative stress and autophagy, which could potentially promote survival.

## 1. Introduction

Doxorubicin (Dox), as represented by anthracycline, is one of the most effective anticancer chemotherapy drugs and has been widely applied to treat a variety of malignant tumors, such as metastatic breast cancer. However, the clinical use of Dox is hindered by its side effects, such as dose-dependent myocardial injury, which is closely associated with cardiomyocytes apoptosis [1], left ventricular dysfunction, and even heart failure in severe cases [2]. Several mechanisms underlying Dox-induced cardiotoxicity are believed to involve oxidative stress caused by free radicals, such as reactive oxygen species (ROS) [3], and also involve reduced levels of antioxidants, especially glutathione (GSH) [4]. Oxidative stress can lead to intracellular lipid peroxidation [5], damaging the cell membranes and other cellular components. In addition, both autophagy and apoptosis play vital roles in the pathogenesis of myocardial injury [6].

At present, the only approved drug by FDA to remit Dox-induced myocardial injury is the cardioprotective agent dexrazoxane (Dex) [7]. Dex is an iron chelator that reduces Dox-induced myocardium toxicity by reducing mitochondrial iron levels and ROS content [8, 9]. However, a variety of side effects are caused by Dex, which hampers the effect of chemotherapy and increases the risk of secondary malignant tumors, especially acute myeloid leukemia and myelodysplastic syndromes. For this reason, European Medicines Agency restricted the use of Dex to adult patients and banned its use in pediatrics [10]. Therefore, it is necessary to study alternative drugs to reduce the cardiotoxicity caused by Dox.

Nuclear factor e2-related factor 2 (Nrf-2) belongs to a family of transcription factors that upregulate a series of gene expressions of antioxidant and detoxification enzymes [11]. In cells under oxidative stress, an autophagic pathway was found to be maintained by a Kelch-like ECH-associated protein 1 (Keap-1)-Nrf-2 feedback loop through p62, a protein encoded by the sequestosome 1 gene (SQSTM1) [12]. To promote the overall survival of cells, Nrf-2 induces transcription of genes that are responsible for detoxifying the ROS and removing damaged proteins [13]. Hence, impaired activation of Nrf-2 leads to amplification or development of inflammation and oxidative stress, which makes it essential to seek strategies to restore the activity of Nrf-2.

**Figure 1:**
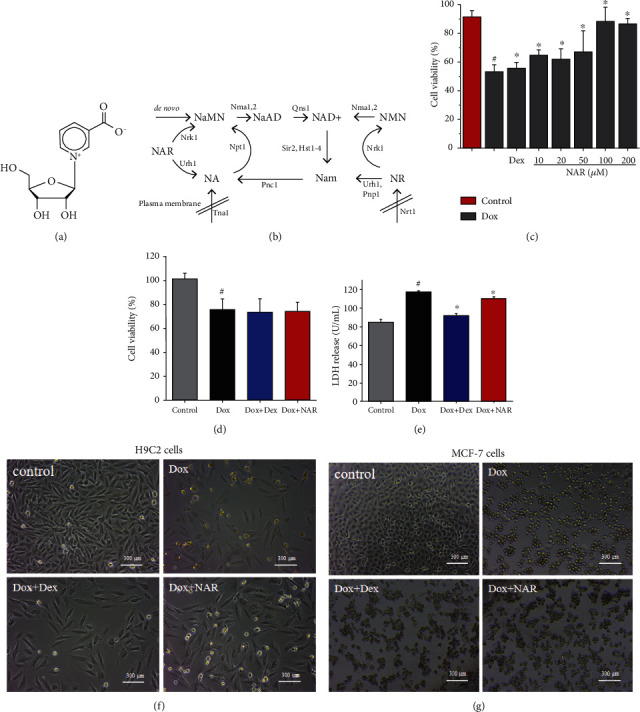
(a) The molecular structure of NAR. (b) Model of NAD^+^ biosynthesis pathways [14].The effect of NAR and Dox administration alone or in combination on cell viability. (c) The cytotoxic effect of different concentrations NAR and Dox alone or in combination on H9c2 cardiomyocytes. (d) The effect of NAR combined with Dox on the cell viability of MCF-7 breast cancer cells. (e) Levels of LDH release. (f) Microstructures of the H9c2 cells under different treatments. Scale bar = 300 *μ*m. (g) Microstructures of the MCF-7 cells under different treatments. Scale bar = 300 *μ*m. The results were analyzed using One-way ANOVA and Student *t*-tests. Data are presented as the mean ± SEM. Significance is indicated as *P* < 0.05. Versus the control, ^#^*P* < 0.05; versus the Dox group, ^∗^*P* < 0.05.

Nicotinic acid riboside (NAR, Figure 1(a)) is an intracellular substance, the precursor of NAD^+^ in eukaryotic cells [14]. Nicotinamide adenine dinucleotide (NAD) is an essential redox carrier, whereas its degradation is a key element of important signaling pathways [15]. NAD supplementation has been reported to contribute to maintaining heart function and immune synergy for treating tumors [16, 17]. Classically known as a hydride-transfer enzyme, NAD^+^ is essential to a variety of diverse biological processes including energy production and synthesis of fatty acids, cholesterol, and steroids [18]. Generally, human cells regulate NAD through biosynthesis using various precursors provided in the diet. Although NAD can be produced from tryptophan through the kynurenic pathway, acid nucleus supply can also be used as a precursor of NAD [19]. NAR (Figure 1(b)) is transported into the cell and is catalyzed to nicotinic acid mononucleotide (NaMN) or niacin (NA), which will then promote the synthesis of NAD^+^ [14].

Doxorubicin-induced cardiomyocyte injury involves a variety of mechanisms, including cardiomyocyte oxidative stress and autophagy [20]. Autophagy can be defined as the process by which cells remove damaged proteins and organelles. Although it is active in all cells, this process is only triggered in response to various types of intracellular stress, so it is selectively activated [21]. Although many studies have described the potential mechanisms of doxorubicin-induced cardiomyocyte injury, there is still a lack of effective cardioprotective methods available. According to our results, it is suggested that Dox-induced oxidative stress and the excessive activation of autophagy might be the key cellular events that lead to Dox-induced cardiotoxicity. NAR supplement mitigates autophagy overactivation and consequently attenuates Dox-induced cardiomyocyte injury through the Nrf-2/p62 pathway.

## 2. Materials and Methods

### 2.1. Cell Culture and Animal Treatment

The H9c2 cell line (ATCC, catalog number CRL-1446) is derived from embryonic rat heart tissue and maintains many cardiomyocyte characteristics. Cells were cultured in Dulbecco's modified Eagle's medium (DMEM), supplemented with 10% fetal bovine serum, 100 U/mL penicillin, and 100 mg/mL streptomycin, at 37°C in a humidified incubator containing 95% air and 5% CO_2_. The medium was refreshed every three days. Cells cultured at about 80% confluence were treated with Dox (5 *μ*M) [22], NAR (5/10/20/100/200 *μ*M), and Dex (20 *μ*M) [23] alone, or in combination, for same incubation times, 24 h [22].

MCF-7 cells (iCell-h129) derived from human metastatic breast cancer were purchased from iCell Bioscience Inc. (Shanghai, China) and cultured in DMEM supplemented with 10% fetal bovine serum,100 U/mL penicillin, and 100 mg/mL streptomycin, at 37°C in a humidified incubator containing 95% air and 5% CO_2_. The medium was refreshed every three days. Cells cultured at about 80% confluence were treated with Dox (5 *μ*M), NAR (100 *μ*M), and Dex (20 *μ*M) alone, or in combination, for same incubation times, 24 h.

The animal experiments were performed on healthy adult female C57BL/6 mice (Sbefo (Beijing) Biotechnology Co., Ltd.) at 8 weeks of age; the mice were normally kept in a pathogen-free environment with free access to food and water. 32 mice were randomly assigned to four groups (*n* = 8 for each group): (i) control group; (ii) Dox treatment group (Dox); (iii) Dox and Dex cotreatment group (Dox+Dex); and (iv) Dox and NAR cotreatment group (Dox+NAR). Dox was intraperitoneally injected on 0, 7, and 14 days, with a dose of 4 mg/kg each time (12 mg/kg cumulative dose of Dox) [24]. NAR (60 mg/kg/day) [25] and Dex (12 mg/kg/day) [7] were intraperitoneally injected every day for a total of 15 times (0 day was initial Dox treatment).

### 2.2. Materials

Nicotinic acid riboside (concentration ≥98%, Maclin, Shanghai, China, batch number: C12093435), doxorubicin (concentration≥99.5%, batch number: HBW200804-3, Hubei Weideli Co., Ltd.), dexrazoxane (concentration ≥99.5%, batch number: HBW200701-3, Hubei Weideli Chemical Technology Co., Ltd.), BCA protein average/concentration detection kit (purchased from Dalian Meilun Biotechnology Co., Ltd.), ATP detection kit, mitochondrial membrane matrix detection kit, TUNEL kit, NAD^+^/NADH detection kit, GSH detection kit, SOD activity detection kit, and lipid oxidation MDA detection kit (all detection kits are purchased from Beyotime Biotechnology Company). All other chemicals and reagents were of analytical grade. DMEM, fetal bovine serum, and cell lysis buffer (10×) were from Meilun Biotech (Dalian, China). Nrf-2, Keap-1, RhoA, ROCK-1, HRP-conjugated GAPDH, LC3B, p62, Beclin 1, Bcl-2, caspase-3, Bax, and IL-4 (antibodies were purchased from ABclonal).

### 2.3. CCK-8 Cell Viability Assay

#### 2.3.1. NAR Concentration Screening

H9c2 cells, pretreated with different concentrations of NAR (5/10/20/100/200 *μ*M) for 24 h, were plated (5000 cells/well) into 96-well plates, examining cell morphological changes by light microscopy. Cell viability was examined using a CCK-8 assay kit (Beyotime Institute of Biotechnology, China). After NAR or Dox treatment, 10 *μ*L CCK-8 was added to each well, and the optical density (OD) value was detected at 450 nm after incubation at 37°C for 2 h.

#### 2.3.2. MCF-7 Proliferation Experiment

Similar to H9c2 cells, MCF-7 breast cancer cells were exposed to Dox (5 *μ*M), Dox (5 *μ*M)+NAR (100 *μ*M) and Dox (5 *μ*M)+Dex (20 *μ*M). Untreated cells were used as controls. Cultured 24 h, examining cell morphological changes by light microscopy. CCK-8 reagent was used to test cell viability to ensure that the NAR therapeutic dose selected by H9c2 cells would not stimulate cell proliferation in MCF-7 cells.

### 2.4. Measurement of the Release of Cardiotoxicity-Related Enzymes

Lactate dehydrogenase (LDH) is an enzyme in the cell that enters the culture medium when the cell is destroyed. Thus, after the 24 h experimental treatment, we collected the supernatant and detected the activity of LDH by using an enzyme labeling instrument (Victor Nivo 3S, PE, USA) according to the specifications of the LDH assay kit (Beyotime Institute of Biotechnology, China).

### 2.5. Cardiac Troponin (cTnI)

The whole blood was centrifuged at 1000*g*, 10 min. The supernatant was then taken, and the serum levels of cTnI were detected by performing ELISA in strict accordance with the manufacturer's instructions (Cloud-Clone Corp. Wuhan, China).

### 2.6. Echocardiography

The cardiac function of the mouse was assessed 2 weeks after the injection of Dox treatment. Mice from four groups were anesthetized with 1% isoflurane inhalation. The experimental animals' cardiac function was measured using an echocardiography system (V6, Vinno China). The ejection fraction (EF) and fractional shortening (FS) were calculated to evaluate cardiac function. Each diameter was obtained from five consecutive cardiac cycles and averaged.

### 2.7. Histological Staining

Mice were sacrificed at 2 weeks following Dox and NAR treatment and the heart tissues were collected. Cardiac tissue was rinsed with phosphate-buffered saline (PBS) and fixed by 4% paraformaldehyde for at least 48 h and embedded in paraffin. The slice was cut into a 5 *μ*m section which was used for hematoxylin-eosin (HE) staining. The images were obtained with a microscope (Nikon, Japan).

### 2.8. Immunohistochemistry Detection

Slice was processed in the same way as HE. Next, the tissue was incubated with 3% H_2_O_2_ at room temperature for 10 min. After that, the tissue was washed with PBS, sealed with goat serum, and then incubated with IL-1*β* antibody overnight at 4°C. Subsequently, the secondary antibody was added to react at 37°C for 15 min. The tissue was next washed with PBS, stained with 0.05% 3,3′-diaminobenzidine (DAB) for 1 min for color development, and counterstained with hematoxylin for 5 min. Finally, after dehydrating and permeating, the sheet was sealed with neutral gum. The number of cells stained positive for IL-1*β* was observed under a microscope (Nikon, Japan).

### 2.9. Measurement of Antioxidant Enzymes and Lipid Peroxidation

Lipid peroxidation is the chain of reactions of oxidative transfer of polyunsaturated fatty acids (PUFAs) to products, recognized as malondialdehyde (MDA), which is regularly evaluated as lipid peroxides. The supernatant of the cellular lysates was collected after treatment and measured according to the manufacturers' instructions. Finally, all collections were tested by using an enzyme labeling instrument (Victor Nivo 3S, PE, USA). The kits for the measurement of malondialdehyde (MDA), superoxide dismutase (SOD), and reduced glutathione (GSH) were purchased from Beyotime Institute of Biotechnology (Beijing, China).

### 2.10. Cellular ATP Content Analysis

Cellular ATP concentrations were assessed with an ATP detection kit (Beyotime Institute of Biotechnology, China). H9c2 cells were lysed through the ATP assay buffer according to the instructions of the manufacturer. ATP content was determined by an enzyme-plate analyzer (Victor Nivo 3S, PE, USA). Unit is nmol/mg protein.

### 2.11. Determination of Intracellular ROS

Dichloro-dihydro-fluorescein diacetate (DCFH-DA), as a membrane-permeable dye, was used to determine whether the reduction in reactive oxygen species (ROS) generation may be an underlying mechanism of cardioprotective effects. The assay was performed according to the manufacturer's guidelines (Beyotime Institute of Biotechnology, China).

### 2.12. NAD^+^ Content and NADPH Oxidase Content Determination Experiment

NAD^+^ and NADPH oxidases (NOX) were extracted from H9c2 cells measured by NAD^+^/NADH Microplate Assay Kit (Beijing Solarbio Science & Technology, China) and NOX Assay Kit (Beijing Solarbio Science & Technology, China) according to the manual, respectively.

### 2.13. Detection of Cardiomyocyte Apoptosis

The apoptosis was evaluated by the terminal deoxynucleotidyl transferase-mediated nick end labeling (TUNEL) staining method, using a kit from Beyotime Institute of Biotechnology, China. Nuclei were stained with DAPI (ZSGB-BIO). In each experiment, cells in at least three random microscopic fields were imaged for the analysis.

Besides, assessment of cardiomyocyte apoptosis using Annexin V-FITC and PI. Cells were seeded at 10^6^ cells per well in a 6-well plate and treated after 24 hours of drug treatment. The assessment of the apoptosis of cardiomyocytes was performed by using an Annexin V-FITC/PI. The assessment of the apoptosis of the cardiomyocytes was performed by using an Annexin V-FITC/PI Apoptosis Detection Kit (Beyotime Institute of Biotechnology, Beijing, China). The stained cells were analyzed by flow cytometry (CytoFLEX A00-1-1102, Beckman, USA) to evaluate cellular damage.

### 2.14. Analysis of Mitochondrial Membrane Potential (*ΔΨ*m)

The mitochondrial membrane potential (*ΔΨ*m) was measured by the fluorescent dye JC-1 staining. *ΔΨ*m was measured using the sensitive fluorescent probe JC-1, a cationic dye of 5,5′,6,6′-tetrachloro1,1′,3,3′-tetraethyl benzimidazole carbocyanine iodide (Beyotime Biotechnology, Shanghai, China). Briefly, stain H9c2 cells with 100 *μ*L) JC-1 at room temperature for 20 min in a dark place. The stained slides were washed three times with PBS and then analyzed immediately with the high-resolution high-speed scanning confocal microscope (Nikon AIR HD25, Japan), which emission at 590 and 540 nm and excitation at 490 nm. For each slide, 5 different fields were selected randomly to acquire images. Cells exhibiting red fluorescence are in normal *ΔΨ*m state. Green fluorescence represented the monomeric form of JC-1 and suggested *ΔΨ*m depolarization. The average intensity of red and green fluorescence was determined and expressed as the proportion of aggregated JC-1 and monomeric JC-1.

### 2.15. Western Blot Analysis

Equivalent protein (10-20 *μ*g) was electrophoresed on sodium dodecyl sulfate-polyacrylamide gels (12%) and transferred to polyvinylidene fluoride (PVDF) membranes. The members were blocked with 5% nonfat milk for 2 h at room temperature. Then, the membranes were probed with specific first antibodies (1:  1000) at 4°C overnight.

After various treatments, the cell samples were collected and lysed by the RIPA buffer; a BCA protein assay was used to analyze the protein concentration. Equivalent protein (20 *μ*L) was electrophoresed on sodium dodecyl sulfate-polyacrylamide gels (use 15% for molecular weight below 60 kDa; use 10% for molecular weight above 60 kDa) and transferred to polyvinylidene fluoride (PVDF) membranes. Then, the membranes were blocked with 5% of milk concentrate (fat-free) at 37°C for 2 hours. After cleansing with Tris-buffered saline with Tween-20 (TBST), the membranes were incubated at 4°C with primary antibodies from ABclonal (Woburn, MA, USA) against Nrf-2 (A0674, 1 : 1000, rabbit), Keap-1 (A17061, 1 : 1000, rabbit), ROCK-1 (A11158, 1 : 1000, rabbit), RhoA (A19106, 1 : 1000, rabbit), LC3 (A19665, 1 : 1000, rabbit), p62 (A19700, 1 : 1000, rabbit), Beclin 1(A7353, 1 : 1000, rabbit), Bax (A20227, 1 : 1000, rabbit), Bcl-2 (A1963, 1 : 1000, rabbit), caspase-3 (A2156, 1 : 1000, rabbit), *β*-tubulin (A12289, 1 : 1000, rabbit), and GAPDH (A19056, 1 : 1000, rabbit). Then, the membranes were incubated with horseradish peroxidase-conjugated secondary antibodies (ABclonal, AC028,1 : 2000, rabbit). Finally, the developed expressions were examined through the enhanced chemiluminescence (ECL) kit (Meilun Biotechnology, China). The GAPDH protein was used as a control. All experiments were evaluated by densitometric analysis with ImageJ software (National Institutes of Health, Bethesda, MD, USA).

### 2.16. Immunofluorescence

For the cultured cells, discard the culture medium, wash twice with cold PBS, first fix with 4% paraformaldehyde for 10 minutes, and wash twice with PBS for 5-10 minutes each time. Permeabilize the membrane for 10 minutes in PBS containing 0.1% Triton X-100 and wash twice with PBS, 5-10 minutes each time. Shake gently several times manually to suck up the liquid. Samples were blocked with 4% goat serum albumin at room temperature for 30 min followed by incubation with primary antibodies against Nrf-2 at 4°C overnight. Then, the samples were washed with PBS and incubated with appropriate secondary antibodies at room temperature for 1 h. Nuclei were stained with DAPI (ZSGB-BIO). Fluorescence images were acquired using the high-resolution high-speed scanning confocal microscope (Nikon AIR HD25, Japan).

### 2.17. Statistical Analysis

Data analysis uses SPSS 19.0 (IBM, Armonk, NY, USA) for the analysis. Data are shown as mean ± standard deviation values. Differences among groups were analyzed with one-way ANOVA analysis, post hoc is SNK, and *P* < 0.05 was considered to be statistically significant.

## 3. Results

### 3.1. NAR Alleviates Dox-Induced Cardiomyocyte Injury in H9c2 Cardiomyocytes but Does Not Affect the Antitumor Effect of Dox on MCF-7 Cells

According to the previous studies, 5 *μ*M Dox concentration was chosen as the treatment dose in the following experiments [26]. To optimize the most effective therapeutic dose of NAR, which is expected to have great protection on H9c2 cardiomyocytes but no obstructive effect of Dox on MCF-7 cells, a CCK-8 assay was employed to quantify cell viability. Compared with other concentrations, 100 *μ*M NAR showed a significant protective effect on Dox-induced cardiomyocyte injury with increased cell viability to a comparable level to the control (Figure 1(c)), and NAR at this dose did not influence the killing effect of Dox on MCF-7 cells (Figure 1(d)). Moreover, Dox+NAR-treated H9c2 cardiomyocytes exhibited normal cell morphology and cell number to that in the control group, but Dox-treated and Dox+NAR-cotreated MCF-7 cells looked similar under light microscopy (Figures 1(f) and 1(g)). Based on these observations, 100 *μ*M Dox was selected for all subsequent experiments. Meanwhile, Dox cotreatment with Dex served as the positive control for all experiments in this study.

Lactate dehydrogenase (LDH) is an important metabolic enzyme in myocardial cells. Therefore, LDH leakage and cell viability generally serve as indicators of cardiomyocyte injury. As mentioned above, cotreatment with NAR increased cell viability compared to Dox-treated cells in a dose-dependent manner (*P* < 0.05, Figure 1(c)). Meanwhile, compared to the Dox group, the addition of NAR significantly decreased Dox-induced LDH leakage (even lower than the Dox+Dex group) detected in the H9c2 culture medium (*p* < 0.05, Figure 1(e)).

### 3.2. NAR Restores Cardiac Function and Architecture *In Vivo*

**Figure 2:**
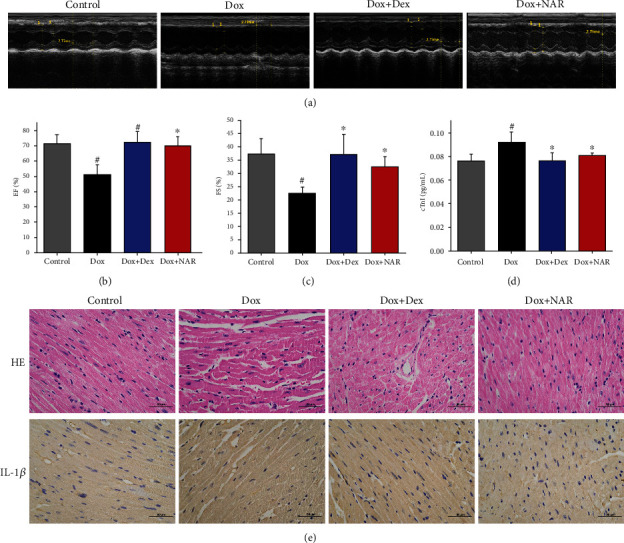
NAR has alleviated Dox-induced cardiac insufficiency. (a) Echocardiogram representative image (*n* = 6), (b) restoring ejection fraction (EF), (c) fractional shortening (FS), (d) cardiac troponin levels(cTnI), and (e) the results from hematoxylin and eosin staining and IL-1*β* immunohistochemistry of myocardial tissue (indicated by brown dots). Data were represented as mean ± SEM. ^#^*P* < 0.05 compared to control; ^∗^*P* < 0.05 compared to Dox.

Cardiac function was assessed by echocardiography following Dox and NAR treatment (Figure 2(a)). As shown in Figures 2(b) and 2(c), Dox administration markedly reduced heart ejection fraction (EF) and fractional shortening (FS) compared to the control group. Cotreatment of Dox and NAR significantly restored EF and FS.

The serum level of cTnI in each group of mice was detected by ELISA. The results showed that the serum level of cTnI in the Dox group was significantly higher than those in the control group (*P* < 0.05), while in the Dox+NAR group, it was reduced to a comparable level to that in the Dex group, suggesting that NAR alleviated cardiac injury caused by Dox (Figure 2(d)).

The morphological changes of myocardial tissue were visualized by HE staining. The untreated animals exhibited normal cellular arrangement and well-organized myocardium architecture in the heart (Figure 2(e)). Dox-treated hearts were characterized by myocardium atrophy, cardiomyocyte disorganization, and myocardial fiber disorder, whereas NAR reversed this phenotype (Figure 2(e)). To add a piece of information on the inflammation state of heart tissue, the proinflammatory factor IL-1*β* was further investigated. The observations showed that Dox-induced an elevated IL-1*β* level, indicating an inflammatory state of myocardial tissue, whereas the addition of NAR eased the situation (Figure 2(e)).

### 3.3. NAR Treatment Preserves the Activities of Antioxidant Enzymes in H9c2 Cardiomyocytes

**Figure 3:**
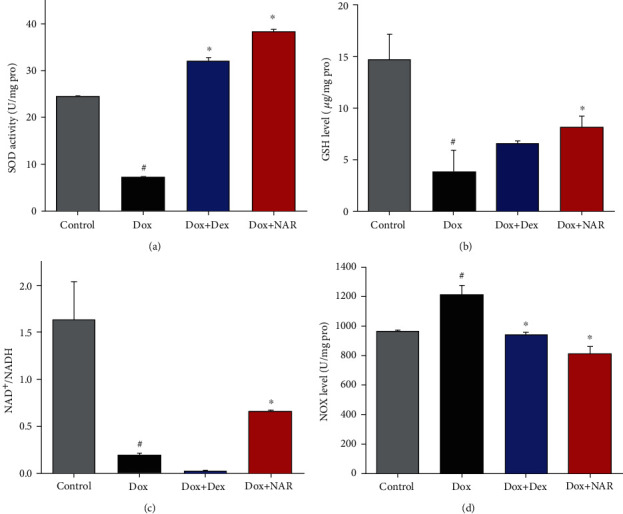
The effect of NAR and Dox administration alone or in combination on the activities of antioxidant enzymes and cell apoptosis in cardiomyocytes. (a) The SOD of NAR and Dox alone or in combination on H9c2 cardiomyocytes. (b) The GSH of NAR and Dox alone or in combination on H9c2 cardiomyocytes. (c) NAD^+^/NADH ratio. (d) NOX contents. The results were analyzed using one-way ANOVA and Student *t*-tests. Data are presented as the mean ± SEM. Significance is indicated as *P* < 0.05. versus the control, ^#^*P* < 0.05; versus the Dox group, ^∗^*P* < 0.05.

SOD and GSH are important antioxidant enzymes that provide the first line of defense against oxidative damage. As shown in Figures 3(a) and 3(b), compared to the control group, the activities of SOD and GSH were downregulated by Dox (*P* < 0.05). Cotreatment of Dox+NAR increased both GSH and SOD levels, which the GSH level was shown to be particularly higher than that in the Dox+Dex group (*P* < 0.05, Figures 3(a) and 3(b)).

### 3.4. NAR Increased the NAD^+^/NADH Ratio and Decreased the NOX Level in the Dox-Treated H9c2 Cardiomyocytes

As shown in Figures 3(c) and 3(d), after Dox treatment for 24 h, the NAD^+^/NADH ratio was reduced, and this was accompanied by elevated NADPH oxidase (NOX) level in H9c2 cardiomyocytes compared to the control group. Notably, these actions were all ameliorated by NAR treatment, in which NAR administration decreased NOX level and lifted the NAD^+^/NADH ratio to some extent (*P* < 0.05, Figures 3(c), 3(d)). Although Dex treatment also led to a decrease in NOX level, the NAD^+^/NADH ratio was not significantly changed compared to the Dox group (Figures 3(c) and 3(d)).

### 3.5. NAR Reduces Cell Apoptosis Induced by Dox in H9c2 Cardiomyocytes

**Figure 4:**
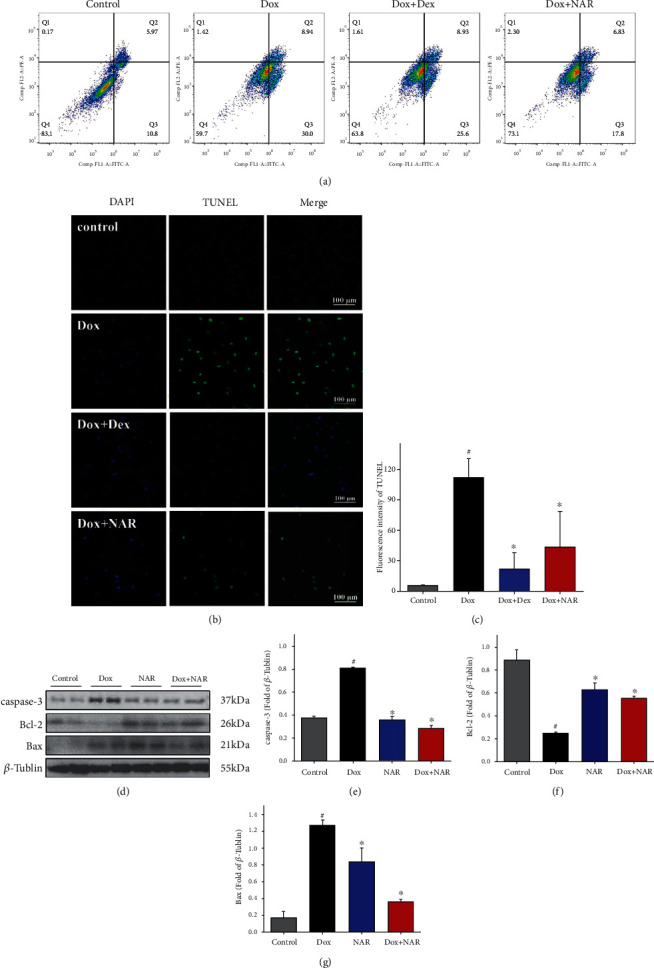
NAR and Dox administration alone or in combination on the cell apoptosis in cardiomyocytes. (a) Flow cytometric dot plots (*x*-axis: Annexin V-FITC staining, *y*-axis: PI staining). (b) The H9c2 cells were analyzed for apoptosis using the TUNEL staining method. The panels display representative histological images (magnification×40). Scale bar = 100 *μ*m. (c) Quantitative analysis of the fluorescence intensity. (d) Expression of cardiac apoptosis proteins in different treatment groups. Densitometric analysis of (e) caspase-3; (f) Bcl-2; and (g) Bax. The figure shows a representative blot for that protein. The values were expressed as fold changes over the level of *β*-tubulin, which served as a loading control. The results were analyzed using one-way ANOVA and Student *t*-tests. Data are presented as the mean ± SEM. Significance is indicated as *P* < 0.05. Versus the control, ^#^*P* < 0.05; versus the Dox group, ^∗^*P* < 0.05.

In addition to cell degeneration and necrosis, cardiomyocyte injury is often accompanied by a significant degree of apoptosis. The degree of apoptosis in the cardiomyocytes was monitored through the quantitative analysis of Annexin V-EGFP/PI staining by flow cytometry analysis. As presented in Figure 4(a), the number of apoptotic cells was higher in the Dox group when compared to the control group (right top, Q2). Pretreatment with NAR lowered the percentage of apoptotic cells. However, the addition of Dex induced more serious apoptosis than Dox treatment, suggesting greater damage to H9c2 cardiomyocytes.

To further investigate cell apoptosis, a TUNEL assay was employed to measure DNA fragmentation in H9c2 cardiomyocytes treated with Dox (5 *μ*M) and NAR (100 *μ*M), alone or in combination. The results showed that the Dox group exhibited a significantly higher ratio of apoptotic cells compared to the control group (Figure 4(b)). While cotreatment with NAR significantly rescued cell death compared to the Dox-treated group (Figure 4(c)). Compared with the NAR group, although the ratio of apoptotic cells was higher than those in the Dex group, they were not significant (*P* > 0.05).

Next, the apoptosis-related proteins were assessed by western blot (Figure 4(d)). It was found that the levels of caspase-3 and Bax were increased and Bcl-2 level was decreased after Dox treatment. Conversely, NAR restored the changes caused by Dox (*P* < 0.05, Figures 4(e), 4(f), and 4(g)). These results indicated that NAR protects against Dox-induced H9c2 cardiomyocytes apoptosis by mediating caspase-3, Bax, and Bcl-2.

### 3.6. NAR Ameliorates Oxidative Stress Induced by Dox Probably by Maintaining the Mitochondrial Function in H9c2 Cardiomyocytes

**Figure 5:**
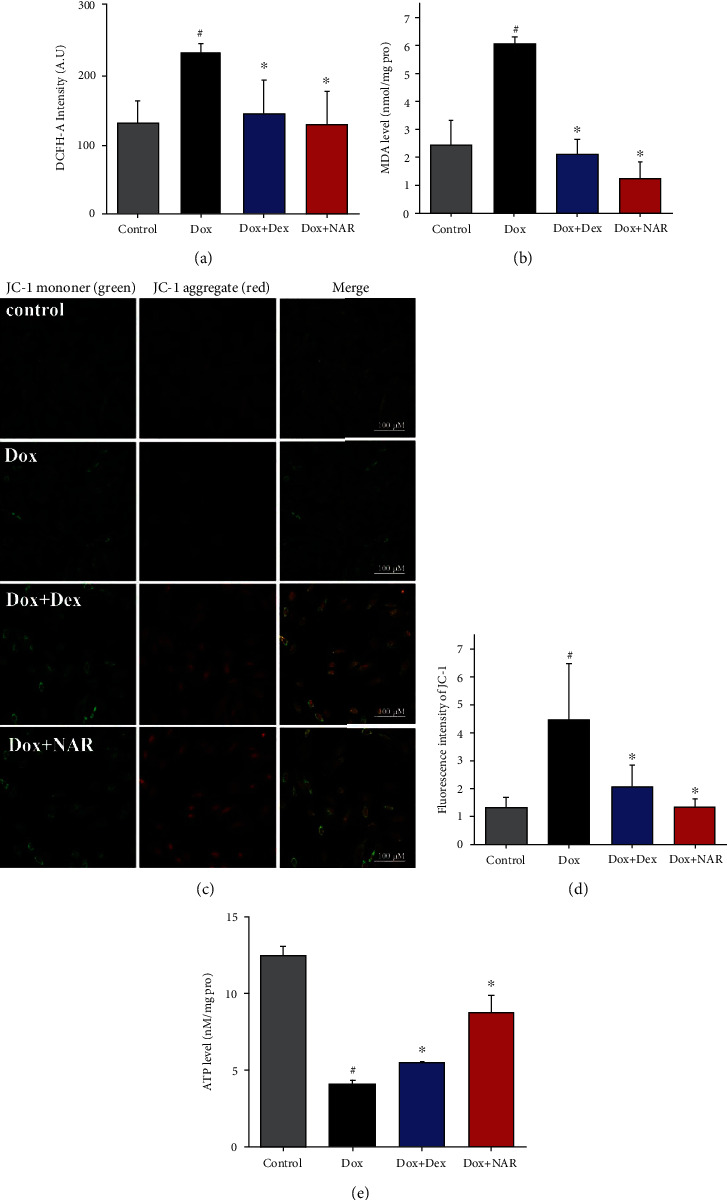
NAR antagonizes doxorubicin-induced cardiomyocyte apoptosis and oxidative stress by protecting mitochondria. (a) The ROS of NAR and Dox alone or in combination on H9c2 cardiomyocytes. (b) MDA level. (c) The images of the effect of doxorubicin, dexrazoxane, and NAR treatment on mitochondrial membrane potential in H9c2 cells (magnification×40). Scale bar = 100 *μ*m. (d) Quantitative analysis of the fluorescence ratio of green fluorescence to red fluorescence is shown. (e) Cellular ATP concentration in H9c2 cells. Data were represented as mean ± SEM. ^#^*P* < 0.05 compared to control; ^∗^*P* < 0.05 compared to Dox.

It is generally accepted that the generation of mitochondrial ROS plays a role in cell death. In cardiac cells, Dox triggers mitochondrial-dependent apoptosis primarily by inducing oxidative stress. Accordingly, Dox-induced ROS production in the H9c2 cardiomyocytes was quantified with DCFH-DA fluorogenic dye. As demonstrated in Figure 5(a), the level of ROS activity increased significantly in the Dox group compared to the control group (*P* < 0.05). Dex and NAR showed similar protection ability against Dox-induced oxidative stress with decreased ROS level observed in both groups (Figure 5(a)).

Furthermore, the activities of lipid peroxidation marker malondialdehyde (MDA) were determined. As shown in Figure 5(b), Dox caused serious oxidative stress in H9c2 cardiomyocytes as indicated by increased MDA level (*P* < 0.05). However, this alteration was ameliorated by NAR treatment (*P* < 0.05).

To investigate whether Dox-induced oxidative stress was linked to mitochondrial dysfunction, JC-1 fluorescence microscopy was employed to examine mitochondrial membrane potential (*Δψ*m). JC-1 fluorescence microscopy showed predominant punctate red fluorescence of mitochondria in untreated cells residing on relatively weak green fluorescence cytosolic background (Figure 5(c)); thus, a high ratio of red to green fluorescence (R/G) serves as the indication of negative mitochondrial membrane potential (*Δψ*m) [27]. In this study, Dox treatment for 24 h caused a significant decrease in red fluorescence in aggregation concomitantly with an increase in green fluorescence (*P* < 0.05, Figures 5(c) and 5(d)). In contrast, the NAR group had a high ratio of R/G. Compared with the Dex group, the NAR group has a higher ratio of R/G, but it is not significant (*P* > 0.05, Figures 5(c) and 5(d)).

In addition, to examine the cytotoxic effect of Dox on mitochondrial function, H9c2 cardiomyocytes were cotreated with either 100 *μ*M NAR or 20 *μ*M Dex for 24 h. The analysis of results showed that Dox treatment at a dose of 5 *μ*M significantly decreased cellular ATP compared to the control (*P* < 0.05, Figure 5(e)). Dox cotreatment with either Dex or NAR significantly ablated this effect (*P* < 0.05, Figure 5(e)).

### 3.7. NAR Attenuated Dox-Induced Excessive Autophagy in H9c2 Cardiomyocytes

**Figure 6:**
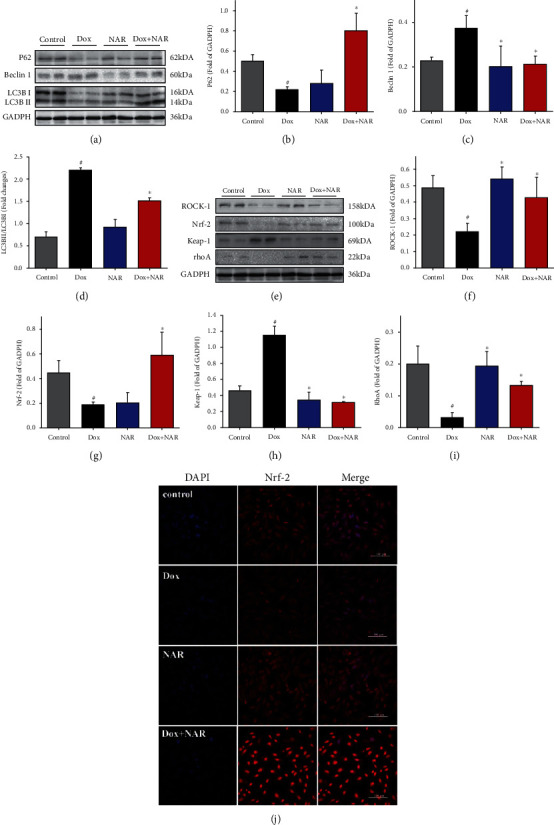
The effect of Dox and NAR on the expression level of oxidative stress proteins and cardiac autophagic proteins after 24 h treatment. (a) Expression of cardiac autophagic proteins in different treatment groups. Densitometric analysis of (b) P62; (c) Beclin 1 and (d) LC3B. (e) Expression of cardiac oxidative stress proteins in different treatment groups. Densitometric analysis of: (f) ROCK-1; (g) Nrf-2; (h) Keap-1; (i) RhoA. The figure shows a representative blot for that protein. The values were expressed as fold changes over the level of GADPH, which served as a loading control. Data were represented as mean ± SEM. ^#^*P* < 0.05 compared to control; ^∗^*P* < 0.05 compared to Dox. (j) The localization of Nrf-2 was examined by confocal microscopy (magnification×40). Scale bar = 100 *μ*m.

To evaluate the effect of NAR on autophagy, the conversion of cytosolic, soluble LC3-I to autophagosomal membrane-attached, lipidated LC3-II, and the key autophagy inducer Beclin 1, was measured by western blotting [28]. As shown in Figures 6(a)–6(d), Dox induced a significant increase in the LC3-II/LC3-I ratio and Beclin 1 protein level as well as a decrease in the level of P62 in H9c2 cardiomyocytes compared to the control group (*P* < 0.05). Cotreatment with NAR reversed the Dox triggered alterations exhibited by reduced LC3-II/LC3-I ratio and Beclin 1 protein level and elevated P62 level (Figures 6(a)–6(d)). The altered LC3-II/LC3-I ratio and Beclin-1 and P62 protein levels suggested an impaired autophagy pathway induced by Dox. The ability of NAR to reverse these changes indicated that NAR attenuated Dox-induced impairment of excessive autophagy in H9c2 cardiomyocytes.

### 3.8. NAR Exerts a Protective Effect against the Dox-Induced Cardiotoxicity Involving Nrf-2 and Its Downstream Signaling Pathways

Western blotting was used to measure the protein levels of Nrf-2, Keap-1, RhoA, and ROCK-1 in H9c2 cardiomyocytes (Figure 6(e)). Dox-treated cells showed decreased protein levels of Nrf-2, RhoA, and ROCK-1 and increased level of Keap-1 protein. Cotreatment with NAR reversed the Dox effect by increasing Nrf-2, RhoA, and ROCK-1 levels and decreasing Keap-1 levels (*P* < 0.05, Figures 6(f)–6(i)). Next, Nrf-2 compartmentalization was visualized by immunofluorescence. NAR induced Nrf-2 translocation into the nucleus; however, it was not observed in Dox and control group (Figure 6(j)).

## 4. Discussions

Doxorubicin (Dox) therapy induces various types of stress to the heart, resulting in cardiomyocyte injury through the induction of oxidative stress, mitochondrial damage, and ultimate apoptosis [21, 29]. Consistent with these studies, we found that cardiomyocytes exposed to Dox undergo more apoptosis with more oxidative stress and mitochondrial dysfunction. Multiple evidence suggests that both apoptosis and autophagy are important factors of Dox-induced heart injury. Our results showed for the first time that exogenous administration of NAR (an NAD^+^ precursor) can protect against Dox-induced cardiotoxicity indicated by restored cardiac function, increased cell viability, improved mitochondrial function, decreased intracellular ROS, and repaired autophagy. In addition, NAR had no interfering effect on the tumor-killing action of Dox.

Dox-induced oxidative stress is mainly governed by reactive oxygen species (ROS) which accumulated to influence the cellular environment, resulting in damages of DNA, RNA, and proteins [11]. Malondialdehyde (MDA) as an oxidation product of lipids plays a central role in oxidative stress injury [30]. Antioxidant enzymes such as superoxide dismutase (SOD) and reduced glutathione (GSH) play major roles in protecting cardiomyocytes against oxidative stress injury [31]. Dox-induced free radicals and consequent lipid peroxidation are important pathogenic events in myocardial cellular injury [32]. The depletion of antioxidants, SOD and GSH concomitant to lipid peroxidation after Dox injections, demonstrates the occurrence of oxidative damage [33]. Treatment with NAR in Dox-induced H9c2 cardiomyocytes resulted in the normalization of SOD and GSH, as well as the inhibition of lipid peroxidation reflected by the reduced formation of MDA.

In addition, redox reactions in cells are enabled by nicotinamide pairs NAD^+^/NADH [11]. Since NAD^+^ plays a vital role in energy metabolism in eukaryotic cells, the maintenance of an optimal NAD^+^/NADH ratio is essential for stabling mitochondrial function, which further preserves ATP contents [34]. Superoxide radical formation includes NADPH oxidases (NOX enzymes) and mitochondria [30]. NOX has been reported to play a major role in promoting oxidative stress. Consistent with our results, NAR reduced intracellular ROS level with increased activities of the endogenous antioxidant enzymes.

NAD^+^-dependent Nrf-2 is the main mediator of cellular adaptation to redox signal [35]. Nrf-2 induces transcription of genes that are responsible for detoxifying the ROS and removing damaged proteins. NAR reduces ROS by enhancing Nrf-2 activity, which then increases antioxidant enzymes (SOD, GSH). As the downstream of Nrf-2, RhoA is a member of the Rho family of GTPases. Upon GTP binding, activated RhoA activates ROCK-1 [36]. ROCK-1 is known to play major roles in a wide range of cellular activities. Thus, the RhoA/ROCK pathway has become a target for the development of drugs for treating cardiovascular diseases [35]. It was reported that Nrf-2 can regulate the RhoA/ROCK signaling pathway to promote cell proliferation and metastasis [37]. Our study showed that NAR could target Nrf-2 to regulate the RhoA/ROCK pathway and then promote the proliferation and motility of H9c2 cardiomyocytes.

Apoptosis is an active process of programmed death through cell death receptors and mitochondria. Apoptosis signals are transferred from death receptors to mitochondria via Bcl-2 family proteins. These proteins include both antiapoptotic proteins (such as Bcl-2) and proapoptotic proteins (such as Bax). Bcl-2 and Bax regulate mitochondrial-dependent apoptotic cell death by modulating the downstream transcription protein caspase-3 [38]. Our results showed that NAR reduced caspase-3 and Bax levels and increased Bcl-2 levels induced by Dox, represented by reduced apoptosis compared to Dox-treated H9c2 cardiomyocytes. During autophagy, Beclin-1 levels are associated with autophagy induction, as it is needed to initiate autophagosome formation that is well characterized by the conversion of LC3-I to LC3-II via phosphatidylethanolamine (PE) conjugation [39]. P62, which is encoded by SQSTM1 [40], is a ubiquitin-binding protein that incorporates into the autophagosome via direct interaction with LC3-II and is degraded in the process of autophagy, and it has been reported that inhibition of autophagy leads to increased P62 levels [41, 42]. In our study, Dox-treated H9c2 cardiomyocytes exhibited increased LC3 II/I ratio and Beclin-1 level and decreased P62 level, indicating excessive autophagy induced by Dox. This observation is consistent with a previous study, in which they showed that Dox inhibits Bcl-2, a negative regulator of Beclin-1, thereby exerting a pathological overactivation of giant cell autophagy, leading to an increase in LC3-II [43]. Excessive autophagy can induce cell death, which is called autophagic cell death [44]. The addition of NAR reversed the alterations in protein levels of LC3 II/I, Beclin-1 level, and P62. Based on available evidence that the regulation of autophagy could attenuate Dox-induced cardiomyocyte injury [45], the reduced cell apoptosis and mitigatory autophagy by NAR treatment suggest that NAR exerts a protective effect on Dox-induced cardiomyocytes injury.

Recently, studies reported the cross-talk between oxidative stress and autophagy through a positive feedback loop involving Nrf-2, in which Nrf-2 activates downstream target genes, including P62, to promote autophagy [46]. To promote the overall survival of cells, Nrf-2 induces gene transcription which is responsible for detoxifying the ROS and removing damaged proteins [13]. Hence, impaired activation of Nrf-2 leads to amplification or development of inflammation and oxidative stress, making it essential to seek strategies to restore the activity of Nrf-2. Under normal conditions, Keap-1 is bound to Nrf-2 and constantly degraded through a ubiquitin-proteasome pathway. In response to oxidative stress, Nrf-2 is released from the Nrf-2/Keap1 complex, through interaction between P62 and the Nrf-2-binding site on Keap1, which leads to Nrf-2 stabilization and activation [47]. Under normal circumstances, the Nrf-2-Keap1-P62 loop is in a dynamic and stable state, maintaining the dynamic balance of cell redox [48]. Once in the state of oxidative stress, the destroyed internal environment will produce excessive ROS, thus initiating autophagy, accelerating the degradation of P62, affecting its binding to Keap-1, making it difficult to activate Nrf-2, and leading to a series of diseases, such as myocardial injury [13]. Consistently, as shown in our results, Dox treatment caused decreased Nrf-2 and P62 levels and increased Keap-1 levels, which indicates that the specific binding of P62 to Keap1 was inhibited, thus affecting the activation of Nrf-2 in H9c2 cardiomyocytes. The addition of NAR completely reversed the Dox-induced dysregulation in proteins involved in apoptosis and autophagy, as well as decreased ROS and apoptosis, and normalized autophagy, which suggests that NAR exerts a protective effect against the Dox-induced cardiotoxicity involving the Nrf-2-Keap1-P62 pathway.

For a better understanding of NAR benefits on Dox-induced cardiotoxicity, mice treated with Dox demonstrated severe tissue damages with distracted tissue architecture, vacuolar degeneration, disorganization, and loss of myofibrils, and NAR injection repaired damaged heart tissue. Autophagy is closely related to inflammation [49]. Therefore, as the proinflammatory factor, IL-1*β* distribution was assessed in Dox-treated hearts with and without NAR. It showed that NAR lessened the inflammatory state in damaged heart tissue caused by Dox.

In summary, the present study demonstrates that Dox can damage cardiomyocytes by inducing oxidative stress, mitochondrial damage, excessive autophagy, and subsequent cell apoptosis, within which Nrf-2 is involved in the regulation of all the above cellular events. NAR protects cardiomyocytes from reducing Dox-induced apoptosis, and the excessive autophagy and oxidative stress ameliorated by NAR involve the Nrf-2/Keap-1/P62 pathway. The outcome of this study suggests that a synergy exists between autophagy and oxidative stress representing a biological mechanism for the presurvival effect of NAR without decreasing the antitumor effect of Dox in MCF-7 cells.

## 5. Conclusion

Dexrazoxane (Dex), in the class of medications known as cardioprotectants, is the only FDA-approved drug used in the management and treatment of anthracycline-induced cardiotoxicity with limitations coming from debatable concerns that Dex might reduce antitumor response rates and increase the risk of secondary hematologic malignancies. Therefore, there is an urgent need to develop new and safer cardioprotective agents. Our findings revealed that treatment with NAR significantly inhibited Dox-induced cardiomyocyte injury by reducing apoptosis rate and oxidative stress, increasing mitochondrial membrane potential, and balancing excessive autophagy. The mechanisms underlying these effects involve Nrf-2-mediated RhoA/ROCK pathway and the Nrf-2/Keap-1/P62 pathway. These findings may provide some new insights for a novel strategy to mitigate Dox-induced cardiomyocyte injury. Therefore, therapeutic strategies that aim to reduce oxidative stress and autophagic activity will presumably be able to ameliorate Dox cardiomyocyte injury, leading to the improved clinical use of Dox in cancer chemotherapy.

## Data Availability

The [DATA TYPE] data used to support the findings of this study are included within the article.

## References

[1] Ma J., Wang Y., Zheng D., Wei M., Xu H., Peng T. (2013). Rac1 signalling mediates doxorubicin-induced cardiotoxicity through both reactive oxygen species-dependent and -independent pathways. *Cardiovascular Research*.

[2] Armenian S. H., Lacchetti C., Barac A. (2017). Prevention and monitoring of cardiac dysfunction in survivors of adult cancers: American Society of Clinical Oncology Clinical Practice Guideline. *Journal of Clinical Oncology*.

[3] He H., Luo Y., Qiao Y. (2018). Curcumin attenuates doxorubicin-induced cardiotoxicityviasuppressing oxidative stress and preventing mitochondrial dysfunction mediated by 14-3-3*γ*. *Food & Function*.

[4] Ghibu S., Delemasure S., Richard C. (2012). General oxidative stress during doxorubicin-induced cardiotoxicity in rats: absence of cardioprotection and low antioxidant efficiency of alpha-lipoic acid. *Biochimie*.

[5] Troyano A., Fernández C., Sancho P., de Blas E., Aller P. (2001). Effect of glutathione depletion on antitumor drug toxicity (apoptosis and necrosis) in U-937 human promonocytic cells. The role of intracellular oxidation. *The Journal of Biological Chemistry*.

[6] Zhang J., Wang M., Ding W. (2020). Resolvin E1 protects against doxorubicin-induced cardiotoxicity by inhibiting oxidative stress, autophagy and apoptosis by targeting AKT/mTOR signaling. *Biochemical Pharmacology*.

[7] Fang X., Wang H., Han D. (2019). Ferroptosis as a target for protection against cardiomyopathy. *Proceedings of the National Academy of Sciences of the United States of America*.

[8] Hasinoff B. B., Patel D., Wu X. (2020). A QSAR study that compares the ability of bisdioxopiperazine analogs of the doxorubicin cardioprotective agent dexrazoxane (ICRF-187) to protect myocytes with DNA topoisomerase II inhibition. *Toxicology and Applied Pharmacology*.

[9] Ichikawa Y., Ghanefar M., Bayeva M. (2014). Cardiotoxicity of doxorubicin is mediated through mitochondrial iron accumulation. *The Journal of Clinical Investigation*.

[10] Tebbi C. K., London W. B., Friedman D. (2007). Dexrazoxane-associated risk for acute myeloid leukemia/myelodysplastic syndrome and other secondary malignancies in pediatric Hodgkin's disease. *Journal of Clinical Oncology*.

[11] Ma Q. (2013). Role of nrf2 in oxidative stress and toxicity. *Annual Review of Pharmacology and Toxicology*.

[12] Deng S., Essandoh K., Wang X. (2020). Tsg101 positively regulates P62-Keap1-Nrf2 pathway to protect hearts against oxidative damage. *Redox Biology*.

[13] Deng Z., Lim J., Wang Q. (2020). ALS-FTLD-linked mutations of SQSTM1/p62 disrupt selective autophagy and NFE2L2/NRF2 anti-oxidative stress pathway. *Autophagy*.

[14] Bogan K. L., Evans C., Belenky P. (2009). Identification of Isn1 and Sdt1 as glucose- and vitamin-regulated nicotinamide mononucleotide and nicotinic acid mononucleotide [corrected] 5'-nucleotidases responsible for production of nicotinamide riboside and nicotinic acid riboside. *The Journal of Biological Chemistry*.

[15] Kulikova V., Shabalin K., Nerinovski K. (2019). Degradation of extracellular NAD(+) intermediates in cultures of human HEK293 cells. *Metabolites*.

[16] Katsyuba E., Auwerx J. (2017). ModulatingNAD+metabolism, from bench to bedside. *The EMBO Journal*.

[17] Li M., Kirtane A. R., Kiyokawa J. (2020). Local targeting of NAD(+) salvage pathway alters the immune tumor microenvironment and enhances checkpoint immunotherapy in glioblastoma. *Cancer Research*.

[18] Bogan K. L., Brenner C. (2008). Nicotinic acid, nicotinamide, and nicotinamide riboside: a molecular evaluation of NAD+ precursor vitamins in human nutrition. *Annual Review of Nutrition*.

[19] Kulikova V., Shabalin K., Nerinovski K. (2015). Generation, release, and uptake of the NAD precursor nicotinic acid riboside by human cells. *The Journal of Biological Chemistry*.

[20] Bartlett J. J., Trivedi P. C., Pulinilkunnil T. (2017). Autophagic dysregulation in doxorubicin cardiomyopathy. *Journal of Molecular and Cellular Cardiology*.

[21] Zilinyi R., Czompa A., Czegledi A. (2018). The Cardioprotective effect of metformin in doxorubicin-induced cardiotoxicity: the role of autophagy. *Molecules*.

[22] Liu M. H., Shan J., Li J., Zhang Y., Lin X. L. (2016). Resveratrol inhibits doxorubicin-induced cardiotoxicity via sirtuin 1 activation in H9c2 cardiomyocytes. *Experimental and Therapeutic Medicine*.

[23] Sangweni N. F., Moremane M., Riedel S. (2020). The prophylactic effect of pinocembrin against doxorubicin-induced cardiotoxicity in an In Vitro H9c2 cell model. *Frontiers in Pharmacology*.

[24] Li M., Sala V., De Santis M. C. (2018). Phosphoinositide 3-kinase gamma inhibition protects from anthracycline cardiotoxicity and reduces tumor growth. *Circulation*.

[25] Cros C., Cannelle H., Laganier L., Grozio A., Canault M. (2021). Safety evaluation after acute and sub-chronic oral administration of high purity nicotinamide mononucleotide (NMN-C®) in Sprague-Dawley rats. *Food and Chemical Toxicology*.

[26] Upadhyay S., Mantha A. K., Dhiman M. (2020). *Glycyrrhiza glabra* (Licorice) root extract attenuates doxorubicin-induced cardiotoxicity via alleviating oxidative stress and stabilising the cardiac health in H9c2 cardiomyocytes. *Journal of Ethnopharmacology*.

[27] Wu Y. P., Zhang S., Xin Y. F. (2021). Evidences for the mechanism of Shenmai injection antagonizing doxorubicin- induced cardiotoxicity. *Phytomedicine*.

[28] Kang L., Liu S., Li J., Tian Y., Xue Y., Liu X. (2020). Parkin and Nrf2 prevent oxidative stress-induced apoptosis in intervertebral endplate chondrocytes via inducing mitophagy and anti-oxidant defenses. *Life Sciences*.

[29] Gu J., Fan Y. Q., Zhang H. L. (2018). Resveratrol suppresses doxorubicin-induced cardiotoxicity by disrupting E2F1 mediated autophagy inhibition and apoptosis promotion. *Biochemical Pharmacology*.

[30] Sies H., Berndt C., Jones D. P. (2017). Oxidative stress. *Annual Review of Biochemistry*.

[31] Zhao L., Qi Y., Xu L. (2018). MicroRNA-140-5p aggravates doxorubicin-induced cardiotoxicity by promoting myocardial oxidative stress via targeting Nrf2 and Sirt2. *Redox Biology*.

[32] Sahu B. D., Kumar J. M., Kuncha M., Borkar R. M., Srinivas R., Sistla R. (2016). Baicalein alleviates doxorubicin-induced cardiotoxicity via suppression of myocardial oxidative stress and apoptosis in mice. *Life Sciences*.

[33] Wang S., Wang Y., Zhang Z., Liu Q., Gu J. (2017). Cardioprotective effects of fibroblast growth factor 21 against doxorubicin- induced toxicity via the SIRT1/LKB1/AMPK pathway. *Cell Death & Disease*.

[34] Dos Santos A. A., López-Granero C., Farina M., Rocha J. B., Bowman A. B., Aschner M. (2018). Oxidative stress, caspase-3 activation and cleavage of ROCK-1 play an essential role in MeHg-induced cell death in primary astroglial cells. *Food and Chemical Toxicology*.

[35] Shimokawa H., Sunamura S., Satoh K. (2016). RhoA/Rho-kinase in the cardiovascular system. *Circulation Research*.

[36] Ko E., Kim D., Min D. W., Kwon S. H., Lee J. Y. (2021). Nrf2 regulates cell motility through RhoA-ROCK1 signalling in non-small-cell lung cancer cells. *Scientific Reports*.

[37] Li D., Wang H., Ding Y. (2018). Targeting the NRF-2/RHOA/ROCK signaling pathway with a novel aziridonin, YD0514, to suppress breast cancer progression and lung metastasis. *Cancer Letters*.

[38] Baechler B. L., Bloemberg D., Quadrilatero J. (2019). Mitophagy regulates mitochondrial network signaling, oxidative stress, and apoptosis during myoblast differentiation. *Autophagy*.

[39] Mizushima N. (2007). Autophagy: process and function. *Genes & Development*.

[40] Luo J., Yan D., Li S. (2020). Allopurinol reduces oxidative stress and activates Nrf2/p62 to attenuate diabetic cardiomyopathy in rats. *Journal of Cellular and Molecular Medicine*.

[41] Klionsky D., Nyfeler B., Murphy L. (2008). Guidelines for the use and interpretation of assays for monitoring autophagy in higher eukaryotes. *Autophagy*.

[42] Mizushima N., Yoshimori T. (2007). How to interpret LC3 immunoblotting. *Autophagy*.

[43] Kobayashi S., Volden P., Timm D., Mao K., Xu X., Liang Q. (2010). Transcription factor GATA4 inhibits doxorubicin-induced autophagy and cardiomyocyte death. *The Journal of Biological Chemistry*.

[44] Wang X., Wang X. L., Chen H. L. (2014). Ghrelin inhibits doxorubicin cardiotoxicity by inhibiting excessive autophagy through AMPK and p38-MAPK. *Biochemical Pharmacology*.

[45] Pan J.-A., Tang Y., Yu J.-Y. (2019). miR-146a attenuates apoptosis and modulates autophagy by targeting TAF9b/P53 pathway in doxorubicin-induced cardiotoxicity. *Cell Death & Disease*.

[46] Tang Z., Hu B., Zang F., Wang J., Zhang X., Chen H. (2019). Nrf2 drives oxidative stress-induced autophagy in nucleus pulposus cells via a Keap1/Nrf2/p62 feedback loop to protect intervertebral disc from degeneration. *Cell Death & Disease*.

[47] Komatsu M., Kurokawa H., Waguri S. (2010). The selective autophagy substrate p62 activates the stress responsive transcription factor Nrf2 through inactivation of Keap1. *Nature Cell Biology*.

[48] Jiang T., Harder B., de la Vega M. R., Wong P. K., Chapman E., Zhang D. D. (2015). p62 links autophagy and Nrf2 signaling. *Free Radical Biology & Medicine*.

[49] Ge Y., Huang M., Yao Y. M. (2018). Autophagy and proinflammatory cytokines: interactions and clinical implications. *Cytokine & Growth Factor Reviews*.

